# Improving the production of the micafungin precursor FR901379 in an industrial production strain

**DOI:** 10.1186/s12934-023-02050-0

**Published:** 2023-03-06

**Authors:** Ping Men, Yu Zhou, Li Xie, Xuan Zhang, Wei Zhang, Xuenian Huang, Xuefeng Lu

**Affiliations:** 1 Shandong Provincial Key Laboratory of Synthetic Biology, Qingdao Institute of Bioenergy and Bioprocess Technology, Chinese Academy of Sciences, Qingdao, 266101 China; 2 Shandong Energy Institute, Qingdao, 266101 China; 3 Qingdao New Energy Shandong Laboratory, Qingdao, 266101 China; 4 University of Chinese Academy of Sciences, Beijing, 100049 China; 5 Institute for Smart Materials & Engineering, University of Jinan, Jinan, 250022 China; 6 State Key Laboratory of Food Science and Technology, Nanchang University, Nanchang, 330096 China; 7 Marine Biology and Biotechnology Laboratory, Qingdao National Laboratory for Marine Science and Technology, Qingdao, 266237 China

**Keywords:** Filamentous fungi, Antifungal agent, Micafungin, Metabolic engineering, *Coleophoma empetri*, Transcriptional activator

## Abstract

**Background:**

Micafungin is an echinocandin-type antifungal agent used for the clinical treatment of invasive fungal infections. It is semisynthesized from the sulfonated lipohexapeptide FR901379, a nonribosomal peptide produced by the filamentous fungus *Coleophoma empetri*. However, the low fermentation efficiency of FR901379 increases the cost of micafungin production and hinders its widespread clinical application.

**Results:**

Here, a highly efficient FR901379-producing strain was constructed via systems metabolic engineering in *C. empetri* MEFC09. First, the biosynthesis pathway of FR901379 was optimized by overexpressing the rate-limiting enzymes cytochrome P450 McfF and McfH, which successfully eliminated the accumulation of unwanted byproducts and increased the production of FR901379. Then, the functions of putative self-resistance genes encoding *β*-1,3-glucan synthase were evaluated in vivo. The deletion of *CEfks1* affected growth and resulted in more spherical cells. Additionally, the transcriptional activator McfJ for the regulation of FR901379 biosynthesis was identified and applied in metabolic engineering. Overexpressing *mcfJ* markedly increased the production of FR901379 from 0.3 g/L to 1.3 g/L. Finally, the engineered strain coexpressing *mcfJ*, *mcfF*, and *mcfH* was constructed for additive effects, and the FR901379 titer reached 4.0 g/L under fed-batch conditions in a 5 L bioreactor.

**Conclusions:**

This study represents a significant improvement for the production of FR901379 and provides guidance for the establishment of efficient fungal cell factories for other echinocandins.

**Supplementary Information:**

The online version contains supplementary material available at 10.1186/s12934-023-02050-0.

## Background

Echinocandin-type antifungal agents are semisynthesized from lipohexapeptides, nonribosomal peptides produced by filamentous fungi [1, 2]. As an inhibitor of *β*-1,3-glucan synthase, echinocandins can potently inhibit the growth of fungal pathogens by blocking the biosynthesis of* β*-1,3-glucan, which is a predominant and specific constituent of the cell wall in most fungi [1, 3]. Therefore, they exhibit pronounced activity against a broad range of *Candida* spp. and *Asperillus* spp., especially against azole-resistant strains [4–7]. To date, caspofungin, micafungin, and anidulafungin have been used as first-line drugs to treat invasive fungal infections [3, 6, 8, 9]. Unlike other echinocandin-type agents, micafungin exhibits excellent water solubility due to the sulfonate moiety originating from the precursor FR901379 [10, 11]. The high water solubility significantly improves the pharmacological efficacy and pharmacokinetic properties [12].

**Fig. 1:**
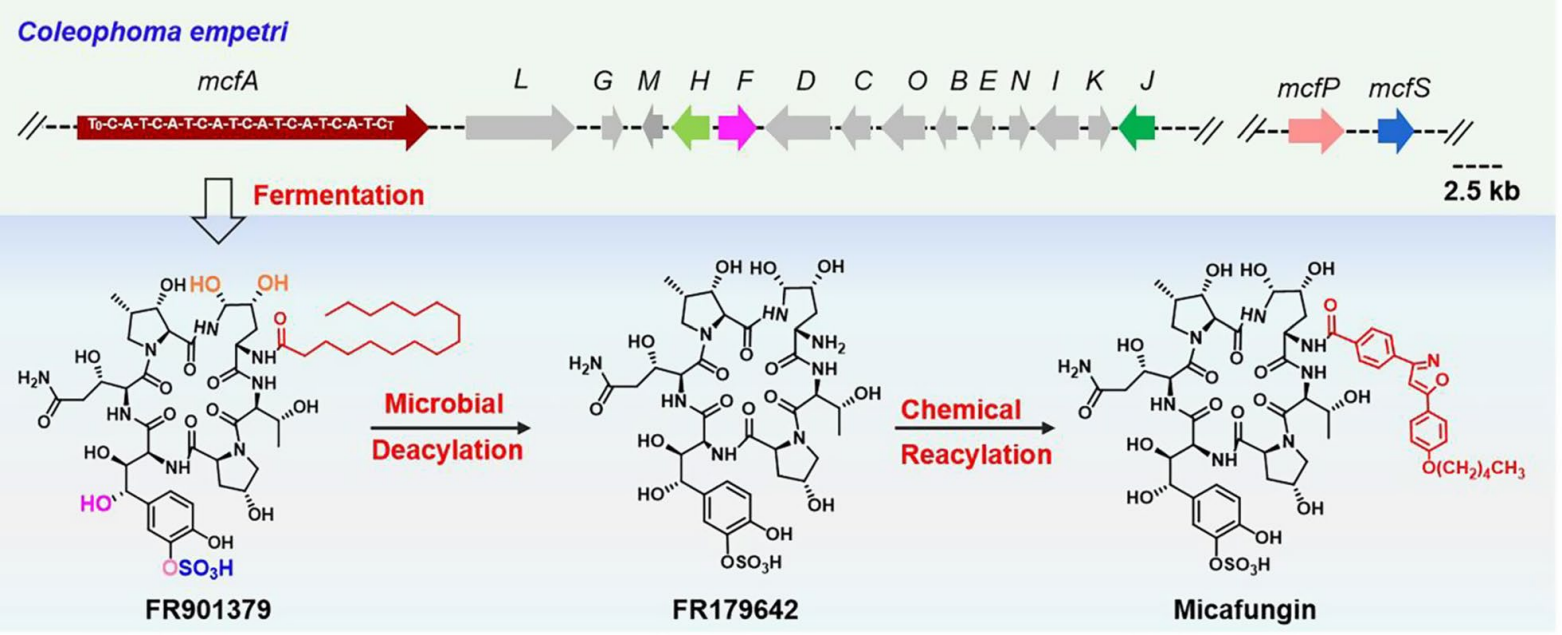
The industrial production process of micafungin and biosynthetic gene clusters of FR901379

Industrial production of micafungin includes three steps: FR901379 is produced through *Coleophoma empetri* fermentation [11], then the palmitic acid side chain of FR901379 is deacylated and substituted with the optimized N-acyl side chain (Fig. 1) [13, 14]. The production of FR901379 is the key step in the manufacture of micafungin. However, the low titer, byproducts with similar structures, and poor mycelium pellet are obstacles to high yield during *C. empetri* cultivation, which increases the production cost and complicates large-scale purification processes [11, 15]. Although mutation breeding and fermentation optimization have been applied to improve the production of FR901379, the above problems remain unsolved [15, 16].

Metabolic engineering provides promising strategies to improve the production of natural products, such as enhancing and optimizing the biosynthesis pathway [17–19], eliminating the competitive pathway [18, 20, 21], and engineering energy homeostasis [22, 23]. Several strategies have been successfully applied to improve the production of echinocandins. By disrupting the key gene for the synthesis of the amino acid building block, the production of the byproduct pneumocandin A_0_ was abolished in the pneumocandin B_0_-producing strain *Glarea lozoyensis* ATCC 20868 [24]. By replacing the proline hydroxylase *gloF* of *G. lozoyensis* SIPI1208 with *ap-htyE* from an echinocandin B-producing strain, the mutants abolished the production of pneumocandin C_0_ and showed an increased titer of pneumocandin B_0_ [25]. For the production of anidulafungin precursor echinocandin B, Min et al. increased the titer of echinocandin B in *Aspergillus delacroxii* SIPIW15 by blocking the biosynthetic pathway of sterigmatocystin [20]. However, there is no report on the metabolic engineering of the FR901379-producing strain because of the absence of a detailed biosynthetic mechanism.

Recently, the biosynthetic pathway of FR901379 in *C. empetri* has been elucidated by gene deletion, enzymatic assays, and heterologous refactoring [26, 27]. The involved biosynthetic genes are distributed in two separate biosynthetic gene clusters (BGCs), the core biosynthetic gene cluster *mcf* and the *O*-sulfonation gene cluster (Fig. 1). The biosynthesis of FR901379 can be divided into three parts: the synthesis of nonproteinogenic amino acids, the assembly of palmitic acid and six building blocks, and the post-modification of cyclized lipohexapeptide. This research progress sheds light on metabolic engineering to improve the production of FR901379 and reduce the accumulation of byproducts. Here, systems metabolic engineering strategies were developed for the FR901379-producing strain *C. empetri* MEFC09, including optimization of the biosynthesis pathway, overexpression of the transcriptional activator, and engineering of self-resistance genes. A more efficient cell factory with a higher FR901379 titer and fewer byproducts was obtained.

## Results and discussion

### Production of FR901379 in *C. empetri* MEFC09

**Fig. 2:**
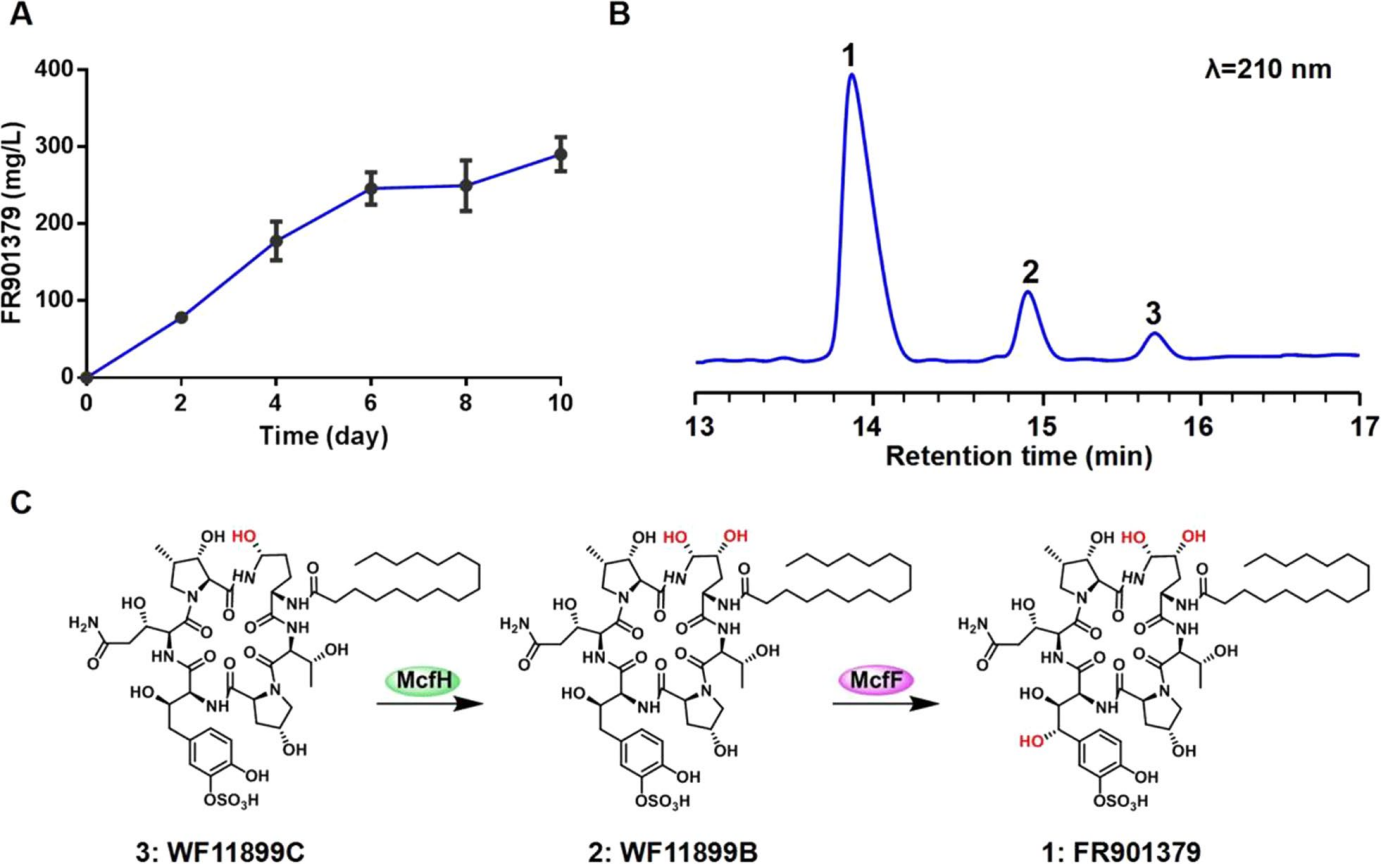
FR901379 production in wild-type *C. empetri* MEFC09. **A** Time course of FR901379 production in *C. empetri* MEFC09. **B** High performance liquid chromatography (HPLC) profile of the extract from *C. empetri* MEFC09; **1**: FR901379; **2**: WF11899B; **3**: WF11899C. **C** The relationship between Compounds **1**, **2**, and **3**

The production capacity of FR901379 of the *C. empetri* MEFC09 parental strain was evaluated in a shake flask. The titer of FR901379 was approximately 300 mg/L when cultured for 10 days, which is still relatively low for industrial manufacture (Fig. 2A). More critically, two analogues accumulated together with the target product, 20% WF11899B (Compound **2**) and 8% WF11899C (Compound **3**, Fig. 2B), which greatly complicate the large-scale purification of FR901379 during the manufacturing of micafungin [11]. The structural differences between FR901379 and these two analogues are the hydroxyl groups located on L-ornithine and 3S-hydroxyl-3'-*O*-sulfonate-homotyrosine (Fig. 2C). Recently, we elucidated the biosynthesis pathway of FR901379, which showed that WF11899B and WF11899C are intermediates prior to hydroxylation in the biosynthesis of FR901379 [27]. The hydroxylations of L-ornithine are catalyzed by the cytochrome P450 (CYP) enzyme McfH and hydroxylation of 3S-hydroxyl-3'-*O*-sulfonate-homotyrosine is catalyzed by the CYP enzyme McfF (Fig. 2C). Therefore, the accumulation of WF11899B and WF11899C might be due to insufficient modification catalyzed by McfH and McfF.

### Optimization of the rate-limiting processes

**Fig. 3:**
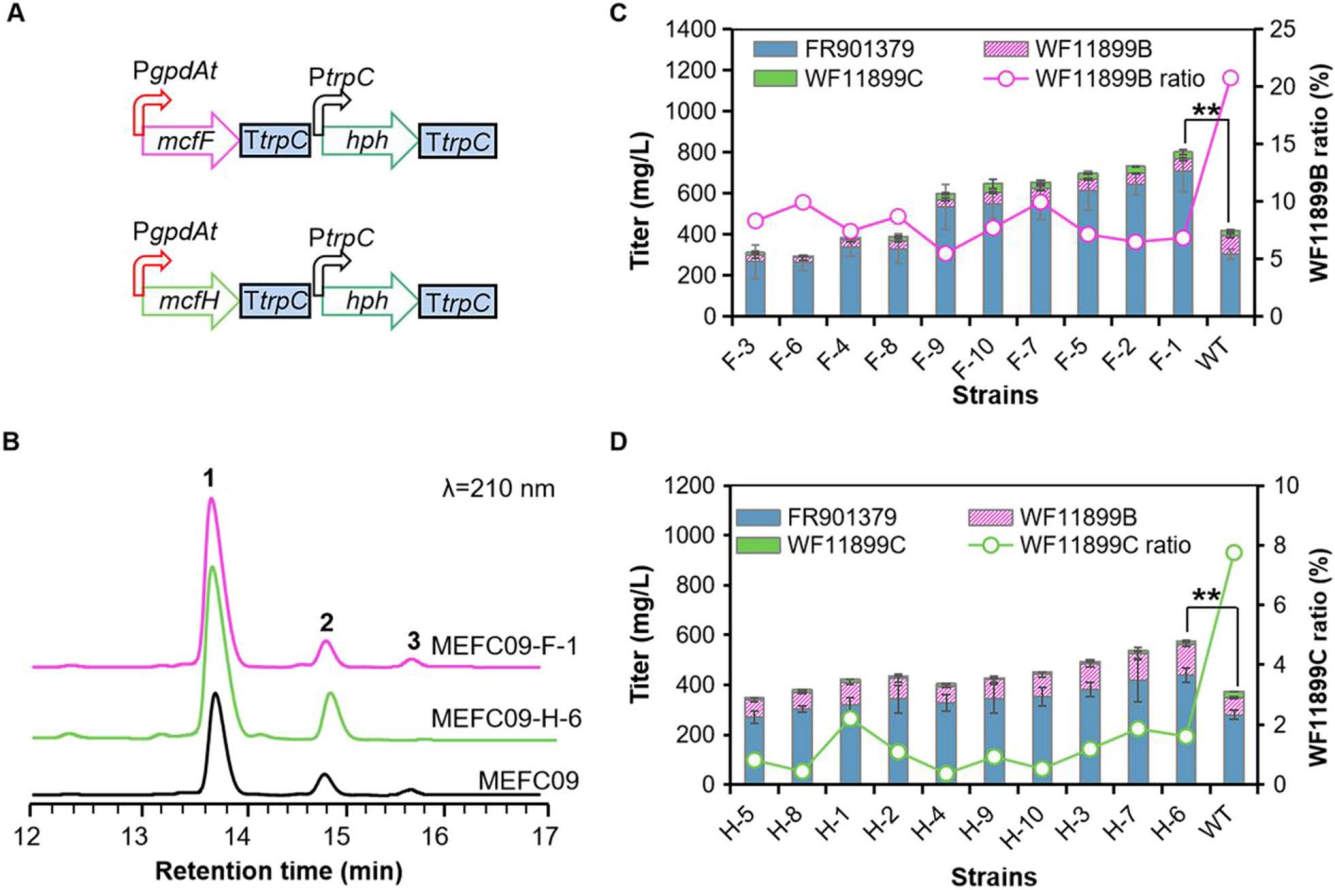
Reducing byproducts through overexpression of *mcfF* and *mcfH* encoding the rate-limiting enzymes. **A** Schematic diagrams of the overexpression of *mcfF* and *mcfH*. **B** HPLC profiles of extracts from MEFC09-F, MEFC09-H, and MEFC09. Titers of FR901379, WF11899B, and WF11899C in the mutant strains of MEFC09-F (**C**) and MEFC09-H (**D**) were quantified; WT: parental strain *C*. *empetri* MEFC09; F: mutant strains MEFC09-F; H: mutant strains MEFC09-H. Statistical analysis was performed by using Student’s t test (**p < 0.01)

To reduce the byproducts (WF11899B and WF11899C) and increase the production of FR901379, the key genes *mcfF* and *mcfH* were overexpressed in *C. empetri* MEFC09 using the promoter of glyceraldehyde-3-phosphate dehydrogenase gene (P*gpdAt*), generating the mutant strains MEFC09-F and MEFC09-H, respectively (Fig. 3A). Because the overexpression cassettes were integrated into the chromosome by random insertion, ten transformants of each construct were randomly selected for shake-flask fermentation evaluation. The ratio of WF11899B was significantly decreased in all transformants of MEFC09-F (Fig. 3B, C). The titers of FR901379 were significantly increased in six transformants. The titer of FR901379 reached 706 mg/L in the best transformant MEFC09-F-1, which was 130% higher than that of *C. empetri* MEFC09 (Fig. 3C). On the other hand, the production of WF11899C was almost eliminated in all transformants of MEFC09-H (Fig. 3B, D). Meanwhile, the titers of FR901379 and WF11899B were both increased to varying degrees (Fig. 3D). These results indicate that the reactions catalyzed by McfF and McfH are indeed the rate-limiting steps in the production of FR901379. In addition, the *O*-sulfonation genes *mcfP* and *mcfS* are also overexpressed in *C. empetri* MEFC09. However, there was no significant difference in the production of FR901379 and other analogues, suggesting that *O*-sulfonation may not be a rate-limiting step in the biosynthesis of FR901379 in *C. empetri* MEFC09 (Additional file 1: Fig. S2).

**Fig. 4:**
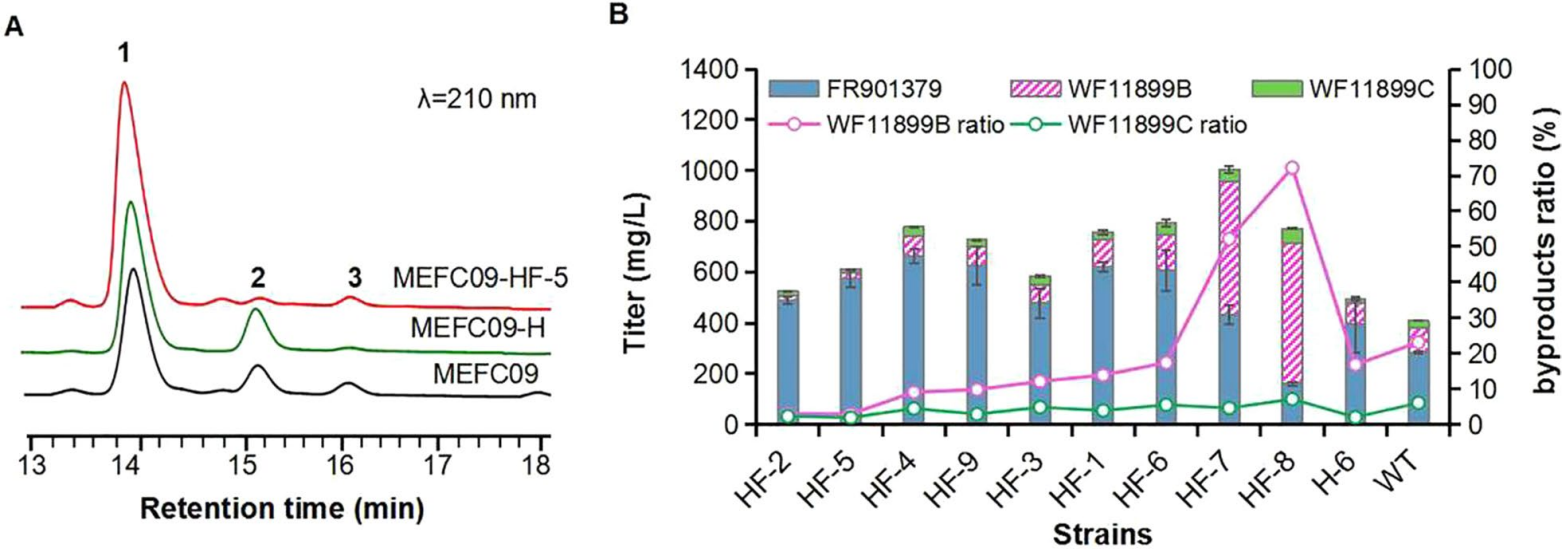
Combinational overexpression of *mcfF* and *mcfH*. **A** HPLC profiles of extracts from strains MEFC09-HF, MEFC09-H, and MEFC09. **B** Titers of FR901379, WF11899B, and WF11899C were quantified in MEFC09-HF; HF: mutant strains MEFC09-HF; H: mutant strains MEFC09-H; WT: parental strain *C. empetri* MEFC09.

To further eliminate WF11899B and WF11899C simultaneously, the expression cassette P*gpdAt*-*mcfF*-T*pgk*-*neo* was introduced into MEFC09-H-6 to reduce the accumulated WF11899B. Nine MEFC09-HF transformants coexpressing *mcfH* and *mcfF* were tested for the production of FR901379, WF11899B, and WF11899C. The results were more complex and variable than overexpressing a single gene because of the random integration of target genes and the incongruity of hydroxylations. In transformants MEFC09-HF-7 and MEFC09-HF-8, the titer of WF11899B was significantly increased and much higher than that of FR901379. This might be due to the imbalance between McfF and McfH caused by the uncertainty of integration sites and copies of expression cassettes [26, 28]. In other transformants, the titers of FR901379 were improved significantly compared to the parental strains MEFC09-H-6 and MEFC09, while the proportions of WF11899B and WF11899C were both reduced (Fig. 4B). In the best mutant MEFC09-HF-5, only very small amounts of WF11899B and WF11899C were detected, and the titer of FR901379 was increased from 284 mg/L to 572 mg/L (Fig. 4A, B). Therefore, an engineered FR901379-producing strain with higher yield and fewer byproducts was constructed by optimizing the rate-limiting steps of FR901379 biosynthesis.

### Functional evaluation of the potential self-resistance genes

**Fig. 5:**
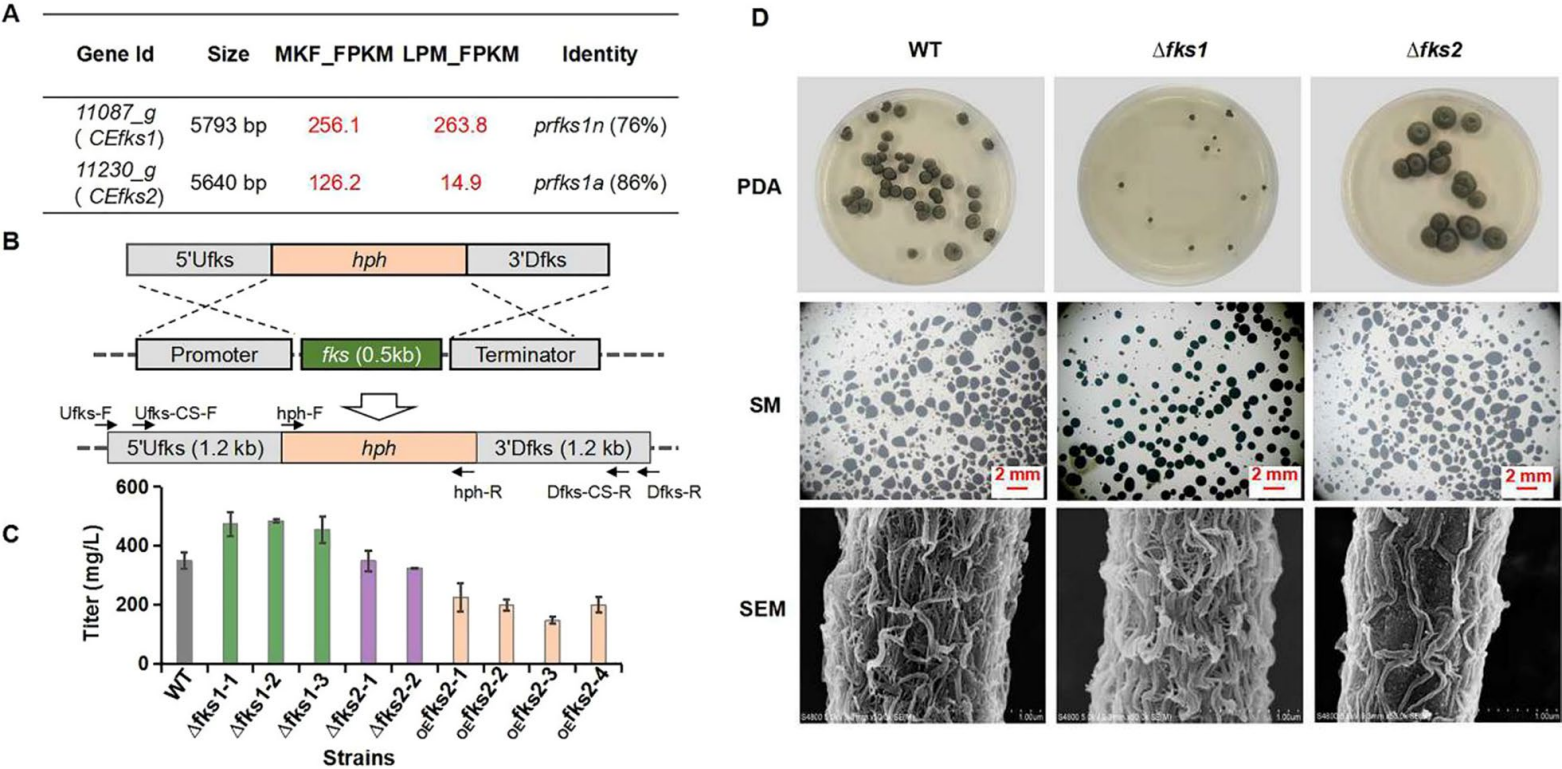
Functional verification of self-resistance genes related to FR901379. **A** The transcription levels and homologous genes of the genes *CEfks1* and *CEfks2*; MKF: FR901379 high-producing condition; LPM: FR901379 low-producing condition; FPKM: Fragments Per Kilobase of exon model per Million mapped fragments. **B** Schematic diagram for the construction of *CEfks1* and *CEfks2* disruption mutants. **C** FR901379 titers were quantified in the *CEfks* disruption strains and *CEfks2* overexpression strains; WT: *C*. *empetri* MEFC09; ∆*fks1*: MEFC09-∆*CEfks1*; ∆*fks2*: MEFC09-∆*CEfks2*; _OE_*fks2*: MEFC09::*CEfks2*. **D** Images of mycelial morphology of the *CEfks1* and *CEfks2* disruption strains; PDA: the strains were grown on PDA medium; SM: stereo microscope images (8 × objective lens) of mycelia of the cultivation broth, all scale bars represent 2 mm; SEM: scanning electron microscopic images of *CEfks* disruption strains

Self-resistance plays an important role in the efficient production of secondary metabolites because of the toxic effects of metabolites on its producer [29]. Malla et al. overexpressed the resistance gene *drrC* in the doxorubicin-producing strain *Streptomyces peucetius* ATCC 27952, and the titer of doxorubicin in the mutant strain was 5.1-fold higher than that of the parental strain [30]. *β*-1,3-glucan synthase is the inhibitory target of echinocandins, indicating that the *β*-1,3-glucan synthase-encoding gene is a self-resistance gene for echinocandin-producing strains [1]. Yue et al. discovered two *β*-1,3-glucan synthase coding genes (*prfks1n* and *prfks1a*) in the echinocandin-producing fungus *P. radicicola* NRRL 12192. *Prfks1n* is essential for cell wall formation and *prfks1a* mainly contributes to protection from echinocandin toxicity [31]. Here, two putative *β*-1,3-glucan synthase coding genes were also found in the genome of *C. empetri* MEFC09 by BLAST analysis. Gene *CEfks1* (*11087_g*) showed 76% identity to *prfks1n*, while *CEfks2* (*11230_g*) showed 86% identity to *prfks1a* (Fig. 5A). Transcriptome analysis results demonstrated that *CEfks1* exhibited high profiles under both high and low FR901379-producing conditions, whereas *CEfks2* only exhibited a high profile under high FR901379-producing conditions (Fig. 5A). Thus, we hypothesize that *CEfks1* is the housekeeping gene responsible for the formation of the cell wall and that *CEfks2* contributes to self-resistance to FR901379.

To verify the effect of *CEfks1* and *CEfks2* on the production of FR901379, these two genes were individually disrupted in *C. empetri* MEFC09 (Fig. 5B). The double-knockout strain could not be obtained. ∆*CEfks1* and ∆*CEfks2* exhibited different growth rates, pellet formation, and cell wall structures (Fig. 5). Deleting *CEfks1* significantly decreased the growth rate on potato dextrose agar (PDA) plates, whereas the colony of ∆*CEfks2* was larger and fluffier than that of *C. empetri* MEFC09 (Fig. 5D). The FR901379 titer of ∆*CEfks1* was 30% higher than that of *C. empetri* MEFC09, which might be caused by improved mycelial morphogenesis (Fig. 5C). The mycelia pellets of ∆*CEfks1* were smaller and more regular than those of MEFC09. However, no obvious change was observed in the strain ∆*CEfks2* (Fig. 5D). Further scanning electron microscopy (SEM) imaging revealed that the structure of the cell wall changed remarkably in both mutant strains ∆*CEfks1* and ∆*CEfks2*. These results demonstrated that these genes both contribute to cell wall formation and affect the morphogenesis and growth of *C. empetri*.

To improve the production of FR901379 by enhancing product resistance, the putative self-resistance gene *CEfks2* was overexpressed in *C. empetri* MEFC09. However, the titer of FR901379 was decreased and accompanied by a slight slowdown in growth (Fig. 5C). This result indicated that the resistance of *C. empetri* MEFC09 to FR901379 was sufficient to protect it from the current concentration of FR901379. Enhancing product resistance is not an effective strategy to improve production. Unlike resistance genes of other natural products, *β*-1,3-glucan synthase plays an important role in fungal cell wall formation. Genetic engineering of *β*-1,3-glucan synthase genes would affect morphogenesis and growth. Therefore, metabolic engineering based on self-resistance genes in echinocandin-producing strains will be more complicated and requires a strict balance between product resistance, growth, and mycelial morphology.

### Improving the production of FR901379 by overexpresing the transcriptional activator McfJ

**Fig. 6:**
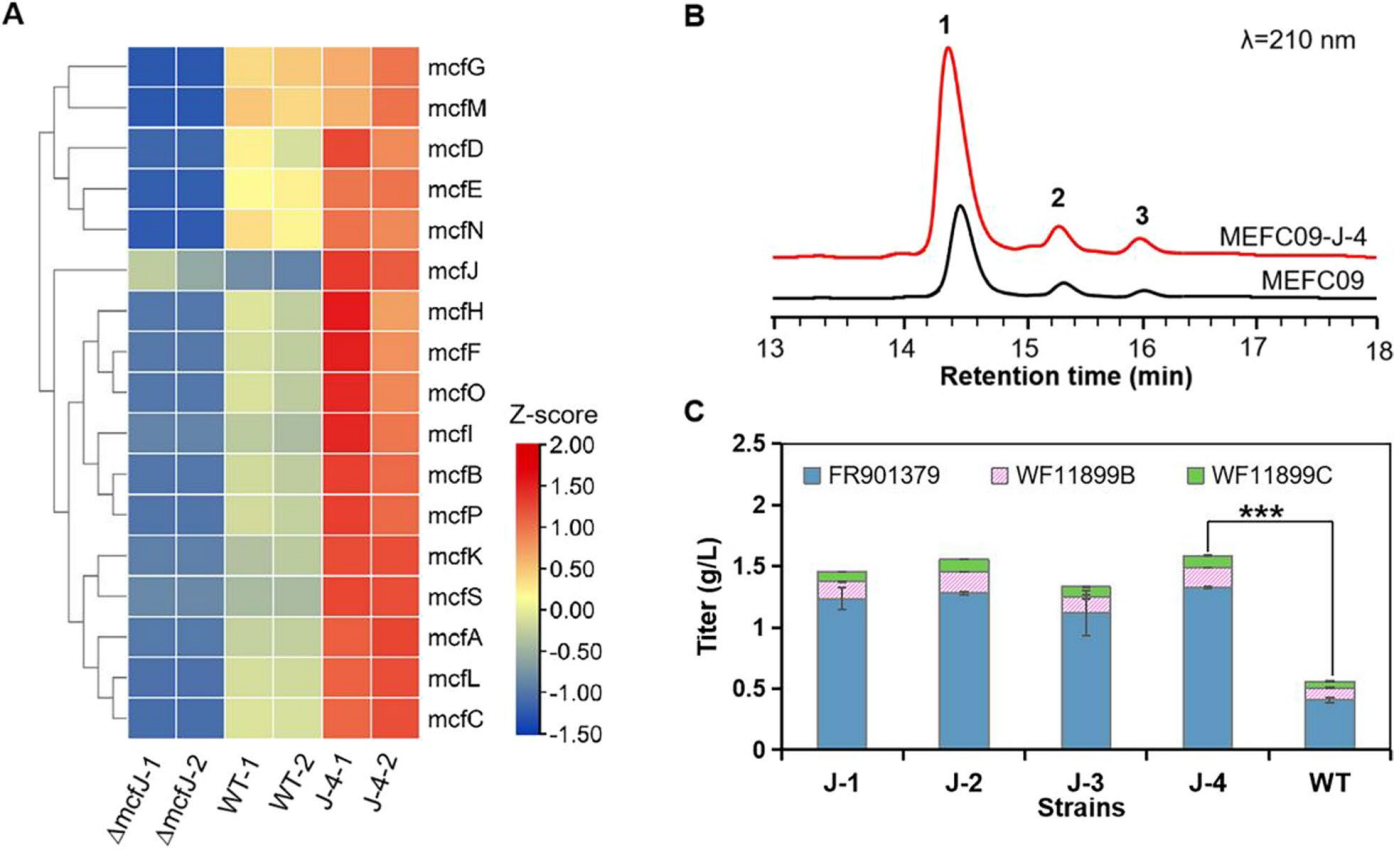
Effects of overexpression of the transcriptional activator *mcfJ* on FR901379 production. **A** Heatmap of the *mcf* gene expression profile of *C. empetri* MEFC09, MEFC09-∆*mcfJ*, and MEFC09-J; WT: *C. empetri* MEFC09; J: mutant strains MEFC09-J. Relative expression levels are shown as a color gradient from low (blue) to high (red). **B** HPLC profiles of metabolites from *C. empetri* MEFC09 and MEFC09-J; **1**: FR901379; **2**: WF11899B; **3**: WF11899C. **C** Titers of FR901379, WF11899B, and WF11899C were quantified in *C. empetri* MEFC09 and MEFC09-J in shake-flask cultures; J: mutant strains MEFC09-J; WT: *C. empetri* MEFC09. Statistical analysis was performed by using Student’s t test (***p < 0.001)

Overexpression of transcriptional activators is an effective strategy to increase the production of secondary metabolites [32, 33]. Overexpressing the specific transcriptional regulator *lovE* significantly improved the production of monacolin J in the industrial strain *Aspergillus terreus* [33]. However, the biosynthetic genes of FR901379 were distributed in two separate BGCs, including the core biosynthetic gene cluster *mcf* and the *O*-sulfonation gene cluster. The regulation of FR901379 biosynthesis is more ambiguous and complicated. The gene deletion and reverse transcription-polymerase chain reaction (RT‒PCR) results showed that *mcfJ* might be a transcriptional activator for the biosynthesis of FR901379 (Additional file 1: Fig. S3). The production of FR901379 was abolished completely in the *mcfJ* deletion mutant. It is consistent with the phenomenon observed in *C. empetri* SIPI1284, deleting *Cehyp* (homologous gene of *mcfJ*) broke the production of FR901379 [26]. The transcriptional levels of the FR901379 biosynthetic pathway, including the genes in the large *mcf* cluster and the separate *O*-sulfonation cluster, were significantly downregulated following *mcfJ* disruption (Fig. 6A, Additional file 1: Table S3).

To further test the function of *mcfJ* and improve the production of FR901379, the expression cassette of *mcfJ* was constructed using the promoter P*gpdAt* and introduced into *C. empetri* MEFC09*.* The transformants harboring the expression cassette were confirmed by genomic PCR and designated MEFC09-J. The results of shake flask fermentation showed that all of the transformants produced significantly higher FR901379 titers than *C. empetri* MEFC09 (Fig. 6B, C). The highest FR901379 titer of 1.32 g/L was achieved in strain MEFC09-J-4, which was threefold higher than that in MEFC09 (Fig. 6C). In addition, the transcription levels of *mcfJ* and other biosynthetic genes of FR901379 were measured by RNA sequencing and were significantly increased in the mutant strain MEFC09-J (Fig. 6A, Additional file 1: Table S3). These results demonstrated that McfJ is indeed a transcriptional activator for the biosynthesis of FR901379. Although the biosynthetic genes were distributed in two separate BGCs, the production of FR901379 was coordinately regulated by McfJ. More importantly, because of this regulatory mechanism, overexpression of *mcfJ* was successfully developed as a very effective metabolic engineering strategy to increase the production capacity of FR901379.

### Construction of a high-yield cell factory for FR901379 by combinatorial metabolic engineering

**Fig. 7:**
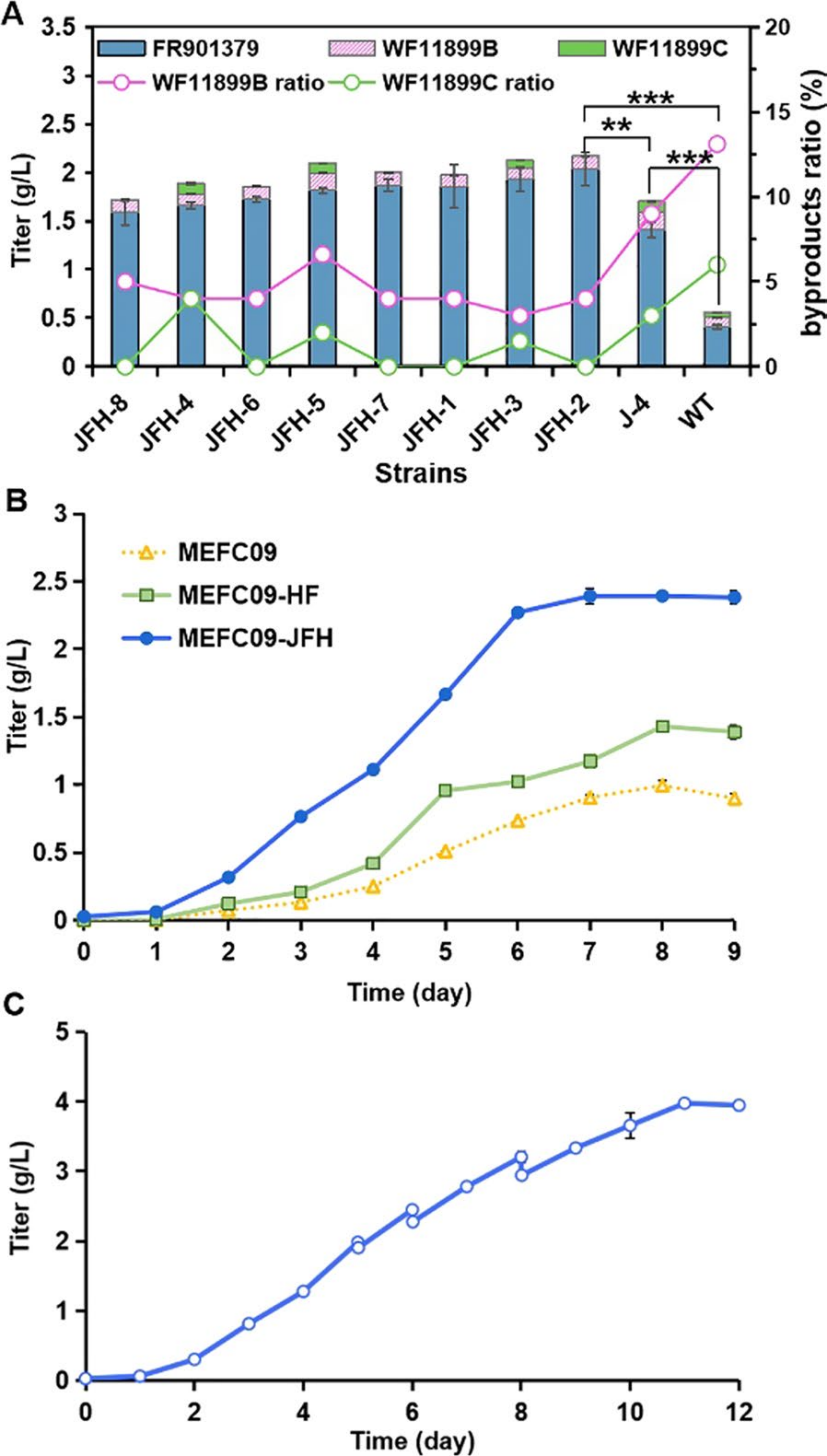
Improving FR901379 titer through combinatorial metabolic engineering and fed-batch fermentation. **A** Titers of FR901379, WF11899B, and WF11899C were quantified in MEFC09-JFH in shake-flask cultures. JFH: mutant strains MEFC09-JFH; J: mutant strain MEFC09-J; WT: *C. empetri* MEFC09. Statistical analysis was performed by using Student’s t test (**p < 0.01; ***p < 0.001). **B** Titers of FR901379 were quantified in the strains MEFC09, MEFC09-HF, and MEFC09-JFH cultivated in a 5 L bioreactor. **C** Titers of FR901379 were quantified in the strain MEFC09-JFH in fed-batch cultivation

Overexpression of CYP enzymes *mcfH* and *mcfF* could significantly reduce the accumulation of byproducts, while overexpression of transcriptional activator *mcfJ* could remarkably increase the production of FR901379. Inspired by the performance of these two strategies, a mutant strain coexpressing *mcfJ*, *mcfF* and *mcfH* was constructed to achieve a superposition of beneficial effects. The expression cassettes of P*gpdAt*-*mcfF*-T*trpC* and P*gpdAt*-*mcfH*-T*trpC* were cointroduced into MEFC09-J-4 to generate the mutant strain MEFC09-JFH. The production of FR901379 was further improved in all engineered strains (Fig. 7A). The highest increase was observed in MEFC09-JFH-2, and the titer of FR901379 reached 2.03 g/L, which was 47% higher than that of MEFC09-J. In addition, the production of byproducts WF11899C was completely abolished in MEFC09-JFH-2, and the WF11899B ratio decreased from 14 to 4%. Therefore, a more efficient cell factory with a higher FR901379 titer and fewer byproducts was constructed by combinatorial metabolic engineering. This is the first transcriptional regulator identified in the biosynthesis of echinocandins, which will serve as a good reference for research on other echinocandins.

### Scale-up production of FR901379 in the 5 L bioreactor

To evaluate the scale-up potential of FR901379 production, batch experiments were carried out in a 5 L bioreactor using engineered strains MEFC09-HF and MEFC09-JFH, with MEFC09 as a control. The titers of FR901379 reached 0.9 g/L, 1.4 g/L, and 2.4 g/L in the strains MEFC09, MEFC09-HF, and MEFC09-JFH, respectively (Fig. 7B). This is significantly higher than those in the shake flask fermentation, which demonstrated the potential for production improvement in the stirred-tank bioreactor. The FR901379 productivity of MEFC09-JFH was much higher than that of MEFC09, MEFC09-HF in the batch fermentation (Additional file 1: Fig. S4). In addition, there was almost no increase in FR901379 production of MEFC09-JFH from Day 6, which was observed from Day 8 in other strains (Fig. 7B). In the fermentation medium MKF for the production of FR901379, *D*-sorbitol is the main carbon source. Further examination of the *D*-sorbitol concentration in the fermentation culture showed that *D*-sorbitol was completely depleted from Day 5 (Additional file 1: Fig. S5A). Therefore, fed-batch fermentation was carried out to achieve higher production of FR901379 using strain MEFC09-JFH. The downward trend in productivity has been significantly slowed. And 4.0 g/L of FR901379 was produced on Day 11 after feeding *D*-sorbitol three times (Fig. 7C), which was the highest production reported to date. To the best of our knowledge, it is substantially higher than the current titer achieved in industrial production .

## Materials and methods

### Strains and cultural conditions

All fungal strains used or constructed in this study are listed in Table 1. These strains were cultivated on PDA medium (pH 5.6, purchased from BD company) at 25 ℃ for 7 days and used for multiplication of the inoculum. The transformants were selected on PDAS (PDA with 0.8 M *D*-sorbitol, pH 5.6) supplemented with appropriate antibiotics as needed, such as 100 mg/L of hygromycin B or geneticin, and cultured for 5–7 days at 30 ℃.

**Table 1:** Strains used in this study

Strains	Characteristics	Reference
MEFC09	Wild type, *Coleophoma empetri*	CGMCC 21058
MEFC09-∆*ku80*	MEFC09 derivate, ∆*ku80*::*neo*	[34]
MEFC09-F	MEFC09 derivate, P*gpdAt*-*mcfF*-T*trpC*-*hph*	This study
MEFC09-H	MEFC09 derivate, P*gpdAt*-*mcfH*-T*trpC*-*hph*	This study
MEFC09-HF	MEFC09-H derivate, P*gpdAt*-*mcfF*-T*trpC*-*neo*	This study
MEFC09-P	MEFC09 derivate, P*gpdAt*-*mcfP*-T*trpC*-*hph*	This study
MEFC09-S	MEFC09 derivate, P*gpdAt*-*mcfS*-T*trpC*-*hph*	This study
MEFC09-∆*CEfks1*	MEFC09-∆*ku80* derivate, ∆*CEfks1*::*hph*	This study
MEFC09-∆*CEfks2*	MEFC09-∆*ku80* derivate, ∆*CEfks2*::*hph*	This study
MEFC09::*CEfks2*	MEFC09 derivate, P*gpdAt*-*CEfks2*-T*pgk*-*hph*	This study
MEFC09-∆*mcfJ*	MEFC09-∆*ku80* derivate, ∆*mcfJ*::*hph*	This study
MEFC09-J	MEFC09 derivate, P*gpdAt*-*mcfJ*-T*pgk*-*hph*	This study
MEFC09-JFH	MEFC09-J derivate, P*gpdAt*-*mcfF-*T*trpC*-*neo,* P*gpdAt*-*mcfH-*T*trpC-neo*	This study

CGMCC: China General Microbiological Culture Collection Center

### Overexpression of target genes in *C. empetri* MEFC09

All plasmids used here are listed in Additional file 1: Table S1. All primers used in this study are listed in Additional file 1: Table S2. The DNA fragments of *mcfF*, *mcfH, mcfP*, and *mcfS* were amplified from genomic DNA of *C. empetri* MEFC09 using respective primer pairs and cloned into vector pXH2-1 at the restriction sites of *Pci* I and *Hind* III, resulting in the plasmids pPM-*mcfF*, pPM-*mcfH*, pPM-*mcfP* and pPM-*mcfS,* respectively (Additional file 1: Fig. S1) [35]. To construct the *mcfJ*-overexpressing mutant strain, *mcfJ* was amplified from the genomic DNA of *C. empetri* MEFC09 using the primers mcfJ-F1/mcfJ-R and cloned into the PU-ZX vector digested with *Xba* I, resulting in the PU-*mcfJ* plasmid (Additional file 1: Fig. S1). The *mcfJ* expression cassette P*gpdAt*-*mcfJ*-T*pgk*-*hph* was amplified from plasmid PU-*mcfJ* using primers PgpdAt-F/hph-R.

All of the expression cassettes were individually introduced into *C. empetri* MEFC09 through (PEG)-CaCl_2_-mediated protoplast transformation [34]. Transformants were selected on PDAS plates amended with 100 mg/L hygromycin B and verified by genomic PCR. To construct the mutant strain MEFC09-HF, the expression cassette P*gpdAt*-*mcfF*-T*trpC* was fused with marker *neo* from pPM-4 by fusion PCR and introduced into MEFC09-H. Similarly, the expression cassettes P*gpdAt*-*mcfF*-T*trpC*-*neo* and P*gpdAt*-*mcfH*-T*trpC*-*neo* were cointroduced into MEFC09-J, resulting in the engineered strain MEFC09-JFH*.*

### Identification of *β*-1,3-glucan synthase

The *β*-1,3-glucan synthase coding genes (*CEfks1* and *CEfks2*) were searched in the genome of *C. empetri* MEFC09 with the sequences of *prfks1n* and *prfks1a* from *Pezicula radicicola* NRRL 12192 as probes [31]. The putative function of the predicted enzymes was confirmed with the online NCBI BLASTP programmer (http://blast.ncbi.nlm.nih.gov). Gene disruption was carried out via homologous recombination as described previously [34]. To knock out the *CEfks1* gene, approximately 1.2 kb of 5' and 3' DNA of the *CEfks1* gene were amplified by PCR from the genome of *C. empetri* MEFC09 using the primer pairs UCEfks1-F/UCEfks1-R and DCEfks1-F/DCEfks1-R and were fused with the marker *hph* by fusion PCR. The gene-targeting cassette was amplified using nest primers and introduced into MEFC09-∆*ku80* by (PEG)-CaCl_2_-mediated protoplast transformation. The mutant strains were verified using the primers UCEfks1-F/DCEfks1-R. A similar strategy was used for the deletion of *CEfks2*. The *CEfks2*-overexpressing strain was constructed as described above.

### Fermentation and HPLC analysis

For the fermentation of all *C. empetri* strains, the fresh mycelia were crushed and inoculated in 50 mL of seed medium MKS (soluble starch 15 g/L, sucrose 10 g/L, cottonseed meal 5 g/L, peptone 10 g/L, KH_2_PO_4_ 1 g/L, and CaCO_3_ 2 g/L; pH 6.5) in 250 mL shake flasks for 2 days at 25 °C and 220 rpm [34]. Then, 5 mL of seed culture was inoculated into 50 mL of fermentation medium MKF (glucose 10 g/L, corn starch 30 g/L, peptone 10 g/L, *D*-sorbitol 160 g/L, (NH_4_)_2_SO_4_ 6 g/L, KH_2_PO_4_ 1 g/L, FeSO_4_·7H_2_O 0.3 g/L, ZnSO_4_·7H_2_O 0.01 g/L, and CaCO_3_ 2 g/L; pH 6.5) and cultivated at 25 °C and 220 rpm for 8 days. The cultivation broth of *C. empetri* MEFC09 and the derived engineered strains was analyzed after cultivated for 8 days. Four-fold volume of methanol was added in each sample and shaken on the vortex oscillator at 2600 rpm for 1 h at room temperature. Three independent experiments were performed for each transformant.

The samples were analyzed by HPLC equipped with a reverse-phase C_18_ column (Agilent, 4.6 × 150 mm, 5 µm) monitored at 210 nm. For HPLC analysis, solvent A was deionized water with 0.05% trifluoroacetic acid and solvent B was acetonitrile with 0.05% trifluoroacetic acid. The following gradient was used at a flow rate of 1 mL/min: 5%-40% solvent B for 3 min, 40%-60% solvent B for 15 min, 100% solvent B for 5 min, and 5% solvent B for 3 min [34]. The amount of FR901379 was quantified based on the peak area.

### Sample preparation for scanning electron microscopy

Sample preparation for SEM was performed according to the method reported previously with modification [36]. The fresh mycelia were collected by centrifugation and washed with phosphate buffer solution (PBS, pH 7.4) three times. The samples were fixed in 2.5% glutaraldehyde fixative for at least 1 h and washed with PBS three times for 10 min each time. Osmium tetroxide was added to the samples for 10 min, then they were washed in PBS three times. The samples were dehydrated with 30%, 50%, 70%, 90%, and 100% ethanol for 5 min each. Next, tertiary butanol was added to the samples for 15 min, and the samples were dried in a freezer dryer and loaded onto specimen stubs. They were coated with gold–palladium before observation under a Hitachi S-4800 SEM Cold Field Emission Microscope (Japan).

### RNA isolation and transcript quantification

Mycelia were collected from the MKF cultures for 2 days and immediately frozen in liquid nitrogen. Total RNA was isolated using a Takara MiniBEST Universal RNA Extraction Kit (Takara, Japan) according to the manufacturer’s protocol. RNA samples were treated with RNase-free DNase I (Takara, Japan) for 15 min to eliminate the genomic DNA. First strand cDNA was synthesized with the PrimeScript™ RT Reagent Kit with gRNA Eraser (Takara, Japan). All of the primers used for RT‒PCR are listed in Additional file 1: Table S2, with the actin gene used as a reference.

The expression profiles of target genes responsible for the biosynthesis of FR901379 of *C. empetri* MEFC09, MEFC09-∆*mcfJ*, and MEFC09-J were compared by RNA sequencing performed by GENEWIZ (Suzhou, China). Then, the corresponding expression levels were obtained by calculating FPKM. The results were illustrated using TBtools based on Euclidean distance calculation [37].

### Cultivation in a stirred-tank bioreactor

For batch fermentation, strains MEFC09, MEFC09-HF, and MEFC09-JFH were cultivated in a 5 L stirred-tank bioreactor containing 3 L of MKF medium. Seed cultures of engineered strains were prepared as described above, then 10% (v/v) seed culture was inoculated into the bioreactor and cultivated at 25 °C for 9 days. The aeration was kept at 1.0 vvm (volume of air under standard conditions/volume of liquid/minute) and the dissolved oxygen (DO) level was controlled at 10% air saturation with an automated cascade to control the stirring rate within a 400–600 rpm range. For fed-batch cultivation, the engineered strain MEFC09-JFH was cultivated for 12 days. After 5 days of incubation, an additional 180 g of *D*-sorbitol was added to the bioreactor on Day 5, Day 6, and Day 8, respectively. Samples were taken every day for HPLC analysis at the same time each day.

## Supplementary Information

**Additional file 1: Table S1.** Plasmids used in this study. **Table S2.** Primers used in this study. **Table S3. **The expression profiles of *C. empetr*i MEFC09 and mutant strains. **Figure S1. **Plasmid maps and cassettes of *mcfF *(A, G), *mcfH *(B, G), *mcfP *(C), *mcfS* (D), and *mcfJ* (E), and *CEfks2* (F). **Figure S2. **Titers of FR901379 were quantified in the mutant strains MEFC09-P and MEFC09-S. **Figure S3. **Functional identification of gene *mcfJ*. **Figure S4. **The concentration of *D*-sorbitol of batch fermentation (A) and fed-batch fermentation (B) in a 5 L bioreactor.

## Data Availability

All data generated and analyzed during this study were included in this manuscript and the additional files.

## Abbreviations

BGCs: Biosynthetic gene clusters
CYP: Cytochrome P450
HPLC: High performance liquid chromatography
P*gpdAt*: Promoter of glyceraldehyde-3-phosphate dehydrogenase gene
PDA: Potato dextrose agar
SEM: Sample preparation for scanning electron microscopyreverse
RT‒PCR: Transcription-polymerase chain reaction
PBS: Phosphate buffer solution
FPKM: Fragments Per Kilobase of exon model per Million mapped fragments
