# Supplementary material for: Improving the production of the micafungin precursor FR901379 in an industrial production strain
Source: Microb Cell Fact. 2023 Mar 6;22:44. doi: 10.1186/s12934-023-02050-0 (PMC9987125; doi:10.1186/s12934-023-02050-0)
Supplement: Supplementary file 1 — Additional file 1: Table S1. Plasmids used in this study. Table S2. Primers used in this study. Table S3. The expression profiles of C. empetri MEFC09 and mutant strains. Figure S1. Plasmid maps and cassettes of mcfF (A, G), mcfH (B, G), mcfP (C), mcfS (D), and mcfJ (E), and CEfks2 (F). Figure S2. Titers of FR901379 were quantified in the mutant strains MEFC09-P and MEFC09-S. Figure S3. Functional identification of gene mcfJ. Figure S4. The concentration of D-sorbitol of batch fermentation (A) and fed-batch fermentation (B) in a 5 L bioreactor. [file 12934_2023_2050_MOESM1_ESM.docx]

**Supporting information**

**Improving the production of the micafungin precursor FR901379 in an industrial production strain**

Ping Men^1,2,3,4^, Yu Zhou^1,2,3,5^, Li Xie^1,2,3,6^, Xuan Zhang^1,2,3^, Wei Zhang^1,2,3,4^, Xuenian Huang^12,3^*, Xuefeng Lu^1,2,3,4,7^*

^1^ Shandong Provincial Key Laboratory of Synthetic Biology, Qingdao Institute of Bioenergy and Bioprocess Technology, Chinese Academy of Sciences, Qingdao 266101, China.

^2^ Shandong Energy Institute, Qingdao 266101, China.

^3^ Qingdao New Energy Shandong Laboratory, Qingdao 266101, China.

^4^ University of Chinese Academy of Sciences, Beijing 100049, China.

^5^ Institute for Smart Materials & Engineering, University of Jinan, Jinan 250022, China.

^6^ State Key Laboratory of Food Science and Technology, Nanchang University, Nanchang 330096, China.

^7^ Marine biology and Biotechnology Laboratory, Qingdao National Laboratory for Marine Science and Technology, Qingdao 266237, China.

* Correspondence to: huangxn@qibebt.ac.cn, lvxf@qibebt.ac.cn

**Table S1** Plasmids used in this study

**Table S2** Primers used in this study

**Table S3** The expression profiles of *C. empetr*i MEFC09 and mutant strains

**Fig. S1** Plasmid maps and cassettes of *mcfF* (A, G), *mcfH* (B, G), *mcfP* (C), *mcfS* (D), and *mcfJ* (E), and *CEfks2* (F).

**Fig. S2** Titers of FR901379 were quantified in the mutant strains MEFC09-P and MEFC09-S

**Fig. S3** Functional identification of gene *mcfJ*.

**Fig. S4** The concentration of *D*-sorbitol of batch fermentation (A) and fed-batch fermentation (B) in a 5 L bioreactor.

**Table S1** Plasmids used in this study

| **Plasmids** | **Characteristics** | **Reference** |
| --- | --- | --- |
| pXH2-1 | Amp^r^, harboring cassettes of *hph*, P*gpdAt*, and T*trpC* | [1] |
| pPM-4 | Amp^r^, harboring cassette of *neo* | [2] |
| PU-ZX | Amp^r^, harboring fragments of P*gpdAt* and T*pgk* | Our lab |
| pPM-*mcfF* | Amp^r^, harboring cassette of *mcfF* | This study |
| pPM-*mcfH* | Amp^r^, harboring cassette of *mcfH* | This study |
| pPM-*mcfP* | Amp^r^, harboring cassette of *mcfP* | This study |
| pPM-*mcfS* | Amp^r^, harboring cassette of *mcfS* | This study |
| PU-*mcfJ* | Amp^r^, harboring cassette of *mcfJ* | This study |

1. Huang X, Lu X, Li JJ: Cloning, characterization and application of a glyceraldehyde-3-phosphate dehydrogenase promoter from *Aspergillus terreus*. *J Ind Microbiol Biotechnol* 2014, 41:585-592.

2. Men P, Wang M, Li J, Geng C, Huang X, Lu X: Establishing an efficient genetic manipulation system for sulfated echinocandin producing fungus *Coleophoma empetri*. *Front Microbiol* 2021, 12:734780.

**Table S2** Primers used in this study

| **Primers** | **Sequence (5'-3')** | **Notes** |
| --- | --- | --- |
| mcfF-FP | caactcatcaatcatcacaacatgctttcagacacgacggc | Amplifying the cassette of *mcfF* |
| mcfF-RP | gatttcagtaacgttaagtggctattccgtccgccttctta |  |
| mcfH-FP | caactcatcaatcatcacaacatggttccatcaatgatctc | Amplifying the cassette of *mcfH* |
| mcfH-RP | gatttcagtaacgttaagtggtcacagggctactttcgatc |  |
| mcfP-F | atttcagtaacgttaagtggatgataaatcttgcaagtc | Amplifying the cassette of *mcfP* |
| mcfP-R | gatttcagtaacgttaagtggctaccgatgaccttcaaggac |  |
| mcfS-F | aactcatcaatcatcacaacatggctttagaccgccagaatgc | Amplifying the cassette of *mcfS* |
| mcfS-R | atttcagtaacgttaagtggctacttcctagctagccaaac |  |
| UCEfks1-F | accaaagctgcacctctcat | Amplifying the upstream of *CEfks1* |
| UCEfks1-R | ctttacgcttgcgatcccgaaacgagatctgagaagatggc |  |
| DCEfks1-F | ctgggttcgcaaagataattgtgccgagtacagatggaaaac | Amplifying the downstream of *CEfks1* |
| DCEfks1-R | gagtccaaatggaaacaccc |  |
| UCEfks1-CS-F | tctcttgatcattggcaaccgg | Amplifying the cassette of *CEfks1*-*hph* |
| DCEfks1-CS-R | tttggctgaaccttgcaaagc |  |
| UCEfks2-F | gcaaggagaggagaacacca | Amplifying the upstream of *CEfks2* |
| UCEfks2-R | ctttacgcttgcgatcccgaattgaaagtcgctgaaaaggtc |  |
| DCEfks2-F | ctgggttcgcaaagataattgccgataagcctgaggatgagg | Amplifying the downstream of *CEfks2* |
| DCEfks2-R | gagaaaagaactgatgacgg |  |
| UCEfks2-CS-F | agtcacgaaggcggacgaaa | Amplifying the cassette of *CEfks2*-*hph* |
| DCEfks2-CS-R | cgccaaggcagctgtggatct |  |
| CEfks2-F | caacaactcatcaatcatcacaatggctcaacctcacgatgcc | Amplifying the cassette of *CEfks2* |
| CEfks2-R | cgtttcacaaaattcttcatttatttattatcggagctttgtaggagtt |  |
| mcfJ-F1 | aactcatcaatcatcacatctagaatgcctatgcctatgtctacc | Amplifying the cassette of *mcfJ* |
| mcfJ-F2 | atgcacttttctcatagtccggg |  |
| mcfJ-R | caaaattcttcatttatttatcaggtcgtcctgcacagct |  |
| PgpdAt-F | aggtacttccattcttactag | Amplifying the cassette of P*gpdAt*-*mcfF*/*mcfH*/*mcfP*/*mcfS*/ *CEfks*2-*hph* |
| hph-R | caattatctttgcgaacccagg |  |
| Ppgk-F | aggtacacttgtttagaggtaataaataaatgaagaattttgtga | Amplifying the cassette of *neo* |
| Tpgk-R | attgcagcgcacaagtcagtc |  |
| E-mcfK-F | cgatgttgacgaccaattcag | RT-PCR for *mcfK* |
| E-mcfK-F | tcccgaaaggtgctttcgtt |  |
| E-mcfI-F | cgtcgccaaaatggtgtcaa | RT-PCR for *mcfI* |
| E-mcfI-R | acagcgacaactttgtcctc |  |
| E-mcfN-F | gtctgttgtaagtcgttgcaga | RT-PCR for *mcfN* |
| E-mcfN-R | ttacgaagtgtgcccacagt |  |
| E-mcfE-F | atcgttgggttgatgagagtg | RT-PCR for *mcfE* |
| E-mcfE-R | aagggttccaaagtctgagt |  |
| E-mcfB-F | cctgatcaaaatgctctgcg | RT-PCR for *mcfB* |
| E-mcfB-R | cgccactggcagtacgtata |  |
| E-mcfO-F | ccagctcatagaaactcccg | RT-PCR for *mcfO* |
| E-mcfO-R | gggtcgtctttcgtgatcga |  |
| E-mcfC-F | tccctggtgcattcttcttct | RT-PCR for *mcfC* |
| E-mcfC-R | agcaagcaggttagctttgt |  |
| E-mcfD-F | tttaaatggcggggttccaa | RT-PCR for *mcfD* |
| E-mcfD-R | ggcgtccttaccaacagtatc |  |
| E-mcfF-F | gcaccaagtaaaccagcatt | RT-PCR for *mcfF* |
| E-mcfF-R | agcagtccaatgtccatacc |  |
| E-mcfH-F | ttggtaatgcagcagcatgt | RT-PCR for *mcfH* |
| E-mcfH-H | ccaatacgtcatctaaagcgg |  |
| E-mcfM-F | attccctgcaaaaggacaacc | RT-PCR for *mcfM* |
| E-mcfM-R | cgtcgtcgatggctcgaaata |  |
| E-mcfG-F | ctcctagctgtacgaacagaac | RT-PCR for *mcfG* |
| E-mcfG-R | cctcggactcggtaatgaaat |  |
| E-mcfL-F | ttgttaggtcgaagtacagcg | RT-PCR for *mcfL* |
| E-mcfL-R | ctcaggtcgaagctcatcac |  |
| E-mcfA-F | ttcgaacctcggagaatcgag | RT-PCR for *mcfA* |
| E-mcfA-R | gctggtcacttccagactaca |  |
| E-mcfP-F | gagctacatgttaatgacccag | RT-PCR for *mcfP* |
| E-mcfP-R | aatgtaaacatcgcggccaca |  |
| E-mcfS-F | ttcgctccagaactcatcgc | RT-PCR for *mcfS* |
| E-mcfS-R | ggaccccatccctcctttac |  |
| E-actin-F | acgagggtttctctcttcct | RT-PCR for actin gene |
| E-actin-R | gctcgttaccaatagtgatgacc |  |

**Table S3** The expression profiles of *C. empetr*i MEFC09 and mutant strains

| Genes | FPKM | | |
| --- | --- | --- | --- |
|  | MEFC09 | MEFC09-∆*mcfJ* | MEFC09-J |
| *mcfA* | 85.39 | 0.41 | 268.77 |
| *mcfL* | 224.54 | 0.7 | 582.17 |
| *mcfG* | 4434.28 | 5.99 | 5729.93 |
| *mcfM* | 609.44 | 0.82 | 843.64 |
| *mcfH* | 888.49 | 0.59 | 2130.84 |
| *mcfF* | 414.66 | 0.27 | 1048.9 |
| *mcfD* | 305.46 | 0.38 | 599.15 |
| *mcfC* | 364.09 | 0.12 | 874.34 |
| *mcfO* | 333.45 | 0.08 | 887.41 |
| *mcfB* | 339.26 | 2.85 | 972.66 |
| *mcfE* | 2201.39 | 1.64 | 3369.79 |
| *mcfN* | 1331.64 | 1.47 | 1932.45 |
| *mcfI* | 712.84 | 0.59 | 2754.18 |
| *mcfK* | 2979.64 | 1.95 | 10349.3 |
| *mcfJ* | 67.71 | 151.3 | 603.63 |
| *mcfP* | 194.73 | 2.05 | 530.02 |
| *mcfS* | 717.05 | 1.07 | 3492.54 |
| actin | 1162.82 | 1089.76 | 1252.14 |

FPKM: Fragments per kilobase of exon per million mapped fragments.

**
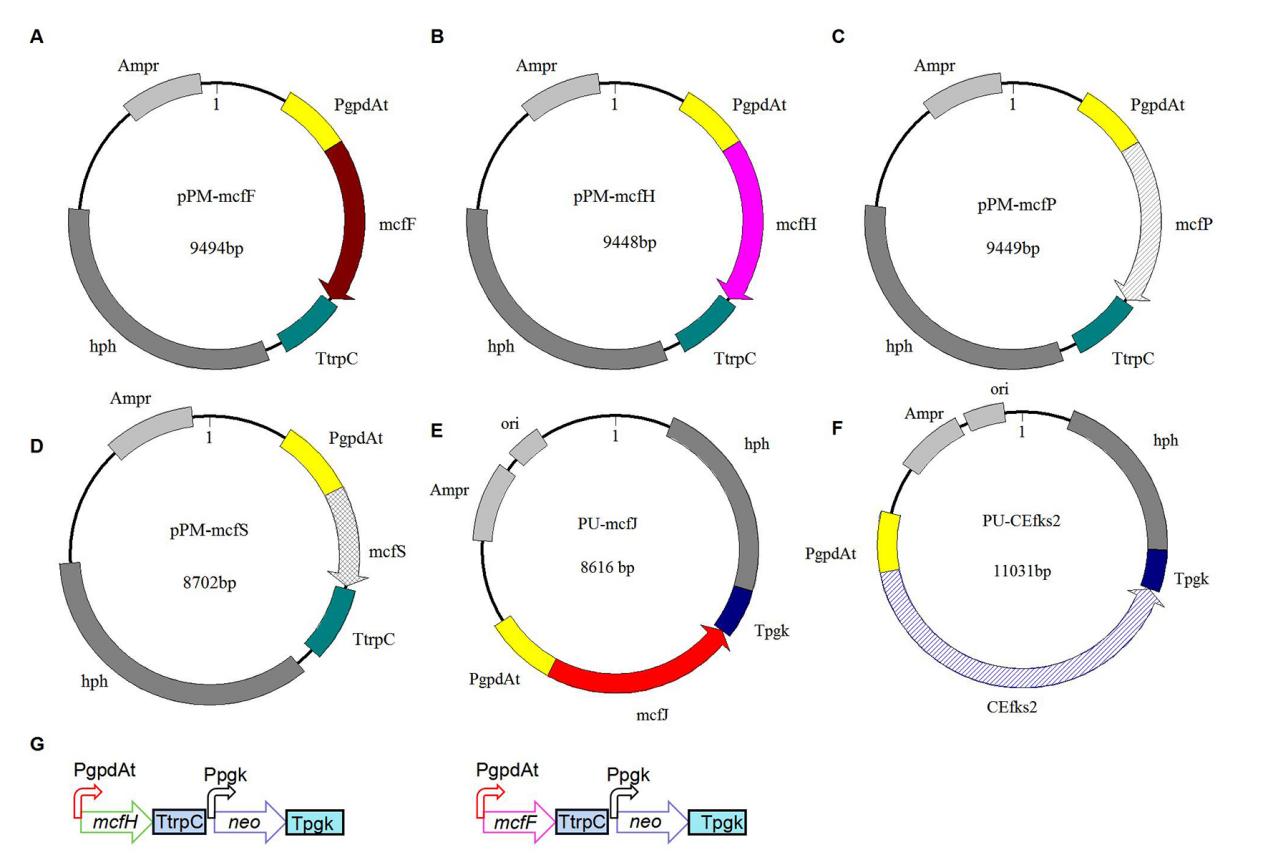
**

**Fig. S1** Plasmid maps and cassettes of *mcfF* (A, G), *mcfH* (B, G), *mcfP* (C), *mcfS* (D), and *mcfJ* (E), and *CEfks2* (F).

**
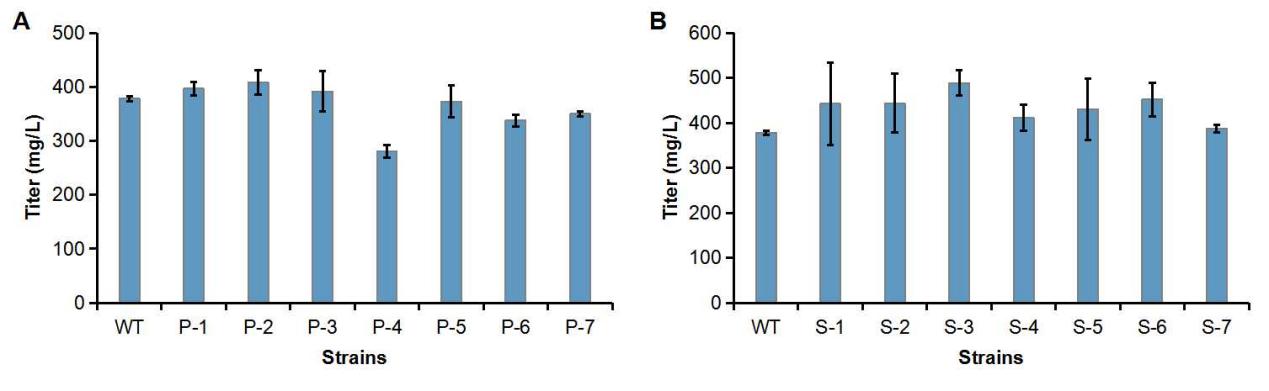
**

**Fig. S2** Titers of FR901379 were quantified in the mutant strains MEFC09-P (A) and MEFC09-S (B); P: mutant strains MEFC09-P; S: mutant strains MEFC09-S; WT: *C.empetri* MEFC09.

**
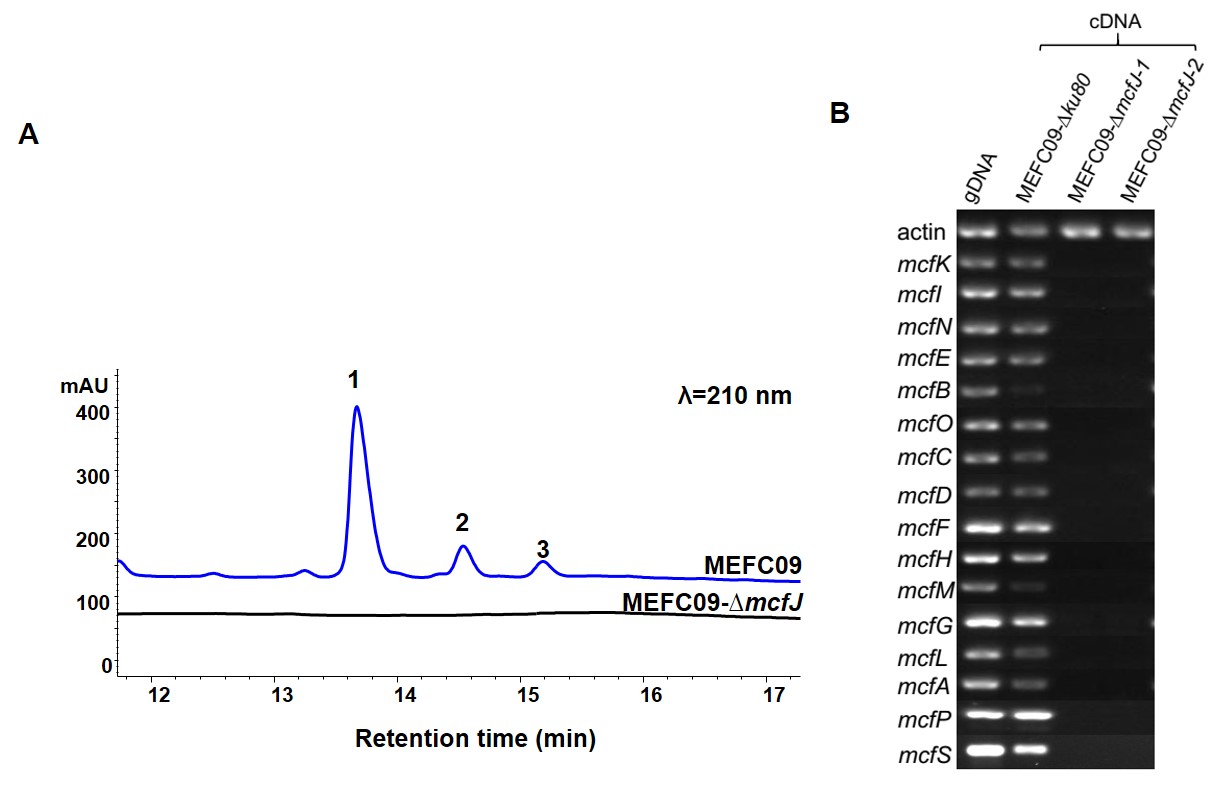
**

**Fig. S3** Functional identification of gene *mcfJ*. (A) HPLC profiles of extracts from *mcfJ* deletion mutant. (B) Comparison of the transcription levels of the genes responsible for the FR901379 biosynthesis, with the actin coding gene as a control.


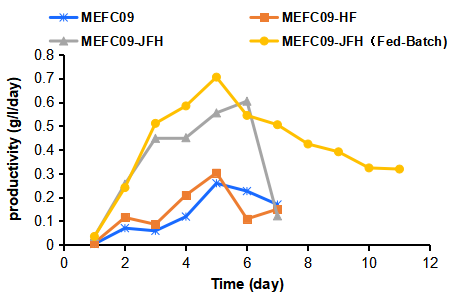


**Fig. S4** The FR901379 productivity of strains MEFC09, MEFC09-HF, MEFC09-JFH in the batch fermentation and MEFC09-JFH in fed-batch fermentation.

**Fig. S5** The *D*-sorbitol concentration in batch fermentation (A) and fed-batch fermentation (B) using a 5 L bioreactor.
